# Theories and practices of disability from the Global South: a critical anthropological perspective

**DOI:** 10.3389/frhs.2024.1261091

**Published:** 2024-04-25

**Authors:** Giorgio Brocco

**Affiliations:** Department of Social and Cultural Anthropology, University of Vienna, Vienna, Austria

**Keywords:** critical disability studies, disability anthropology, Global South, disability categories, gender and disability

## Abstract

“Critical disability studies” (CDS) is an interdisciplinary field of research that examines social, political, economic, racial, gendered and historical constructions of bodily non-normativity across different geopolitical areas and scales. Despite its diverse and multiple contributions and objectives, current research in critical disability studies has been described as mainly focusing on disability issues in the Global North and as having universalizing tendencies. In this context, intersubjective perspectives and empirical data offered by ethnographic works in medical and disability anthropology and related disciplines have been either in accord or tension with the broader field of CDS. On the one hand, this review article illustrates the many ways anthropologists have adopted various research perspectives to explore bodily non-normativity outside settings in the Global North. On the other, it shows the importance of research by anthropologists working on topics related to disability, as well as their recent fruitful collaborations with CDS scholars and approaches. By exploring these epistemological and empirical entanglements, this paper concludes that deeper engagements between CDS and anthropology, as well as a more thorough focus on the ethnographic analysis of bodily non-normativity, can open new creative routes for the analysis of disability in various world contexts.

## 1 Introduction

Writing, analyzing, and examining diverse forms of bodily difference^1^ and their sociocultural representations and manifestations worldwide is a challenging endeavor (1). Critical disability studies (CDS), or critical disability theory (CDT)^2^, includes interdisciplinary approaches to analyze disability as a socio-political, historical, and cultural phenomenon that is shaped by symbolic and sociocultural structures, political ideas, literary representations, narratives and practices in various world settings (2–5). Scholars in this broad field strive to denaturalize disability and question how it is defined in terms of the Global North (6). The field of critical disability studies is an interdisciplinary arena not only for understanding bodily “alterity” but also advancing forms of activism and advocacy that can expose historical and sociocultural norms, prejudices and biases that categorize certain bodily characteristics as non-normative (7, 8). Further complementary focuses include the sociocultural, political, and economic contexts that produce stigma and marginalization as well as agentic practices of resistance against these attitudes. Therefore, CDS challenges white and mestizaje-based ableist ideas of disability politics in societies, institutions and ideologies (9, 10). While sharing certain methodologies and perspectives with the “social model of disability”, some scholars in CDS criticize previous approaches for their liberal perspectives, narrow focus on physical disabilities, and emphasis on Global North contexts and concepts like “independent living,” as well as their entanglements with neoliberalism, masculinity, white supremacy and somatophobia (1, 2). Moreover, previous models and scholarly trends pay little attention to racial and linguistic differences in the analysis of disability issues (1, 11).

A primary research goal in CDS is therefore to foster collaboration and networks of solidarity that include “marginalized” individuals who may not identify as disabled but experience forms of “devaluation” or pathologization (7). Accessibility issues are another significant concern for people with non-normative bodies^3^, scholars and activists in CDS (1). The broader aims of researchers in this field include examining the social, economic, political, and historical constructions of disability and its various identity politics (13), as well as the emotional and social impacts that categorizations have on individuals with non-normative characteristics (1, 14). Intersectionality is thus one epistemic approach they adopt to explore how disability—and ideas, discourses and narratives about it—overlap with race, class and gender (15).

Before the emergence of CDS, various analytical approaches had addressed issues of stigma, vulnerability, marginalization, non-normativity, and exclusion. Some scholars, influenced by the “social model of disability,” attributed oppression against people with disability to social, economic, and political power dynamics (1, 6). Others have paid attention to the sociocultural and economic mismatches between non-normative bodies and societal expectations (13). Such perspectives have been also enriched by their intertwinement with other theoretical questions. Queer perspectives, feminist theories, and post- and decolonial approaches have offered valuable theoretical and empirical points of view for analyzing disability as a political and social phenomenon, as well as for unraveling the intricate connections between non-normative bodies, racial issues, gender divisions, class relationships and histories of domination and colonialism (10, 11, 16). Revolving around the ability/disability dichotomy, categorizations and representations of disability and able-bodiedness in fact permeate all aspects of social life and perpetuate ableist social norms (7).

Beside the emergence of “crip theory” for the analysis of the intertwined issues of disability, sexuality and queerness (17), new points of view for the study of disability have emerged in CDS, including Disability Critical Race Studies, Black Disability Studies, and Indigenous Post-Colonial Theories (1). On the one hand, these perspectives describe the intersectional issues related to living with a disability in exclusionary, racist and ableist societies (9, 15, 18). On the other, they focus their attention on indigenous, moral-spiritual ideas, practices and discourses about disability: for example, in Māori and Native American sociocultural groups and networks (1, 19) as well as Ubuntu perspectives from South Africa and other indigenous conceptualizations from the African continent at large (20–24). Further methodological and theoretical entanglements include those between CDS and post- and decolonial theories, in which matters of coloniality, decoloniality and neocolonialism have been explored and connected to the (re)production and (re)shape of disability (10, 17, 25–27) and beyond Global North epistemics (2, 9, 28, 29). Over the years, CDS scholars have also critiqued the global disability movement and the “social model of disability” for their close alignment with dominant Global North representations of disability (30–33).

Within this epistemological scenario, disciplines like Social and Cultural Anthropology have since the 1990s also examined disability and non-normative bodies in many Global South sociocultural contexts^4^ and from various contingent and divergent theoretical perspectives (35–40). This anthropological interest in disability has developed through both tensions and engagements with theories from disability studies (DS). In the early 2000s, DS researchers even seemed reluctant to incorporate anthropological insights and methodologies (41). Kasnitz and Shuttleworth (42) have attributed this issue to a lack of anthropological expertise, which limited scholars' capacity for critical and comparative study of disability in diverse sociocultural contexts. Despite these critiques, however, a group of interdisciplinary scholars (42, 43) started to emphasize how anthropological methods and a cross-cultural, ethnographic-based, comparative approaches could greatly benefit studies in what was then called the DS arena (42, 44).

At the same time, the relationship between anthropology and DS was hampered by the hesitance of anthropologists to approach discussions and theories in Disability Studies. Although anthropologists were encountering disabled interlocutors and talked about their socio-cultural positions and relations within their field (26), they initially considered disability a secondary topic of research. Staples and Mehotra (26) explain this phenomenon by highlighting the lack of mutual conversation and intellectual debate between anthropologists and scholars in “the realm of disability studies” as well as DS researchers' tendency to seek to define disability according to universal analytical categories. Despite these epistemic disagreements, between 1995 and 2006 various anthropologists started to generate ethnographies and to collect data about people with disability and disability movements/groups in both the Global North and South (35, 45–50).

Over the last 15–20 years, anthropologists’ interest has grown significantly, especially within the initial space offered by Medical Anthropology (26, 48). Despite this, many DS scholars have continued to warn of the risk of (indirectly) re-medicalizing forms of non-normative bodies. On the other hand, anthropologists have outlined the disadvantages of affirming new types of identity categories related to disability like those of gender and ethnicity. For these interconnected reasons, anthropological research on disability have been considered “rather scattered and fragmented” (26), even though anthropologists have continued researching experiences of bodily non-normativity, as demonstrated by, for example, recent research on disability in India (51, 52). The mutual tensions and engagements between CDS and DS, on the one hand, and Anthropology, on the other, still exist in present-day academic landscapes. According to Staples and Mehotra (26), such divergences can be seen as creative and generative of new methodological and theoretical approaches. This mutual enrichment can be inscribed into the initial biases inherent to both these two arenas: the universalizing categorizations applied by scholars in DS and CDS and the anthropological focus on long-term and “patchworked” ethnographic research about local notions, practices and ideas of bodily difference and non-normativity (26). At the same time, other scholars (53–56) have called for the constitution of a Disability Anthropology which not only draws on and contributes to debates in CDS but “engages the distinctive theoretical concerns and methodological approaches of transdisciplinary critical disability studies” (57).

Recent research in anthropology has highlighted that disability, in its plurality, is a relational category shaped by sociocultural, political, economic, geographical, racial, gender, and historical differences and dynamics (37–39, 58). Connections to local, cosmological, moral and religious ideas represent further forms of relationality that foster ideas of bodily non-normativity, as in the case of childhood disability and practices of selective reproduction in Vietnam (59). To emphasize its relational character, anthropologists have also pointed out that the term “disability” has never appeared outside Global North/Euro-American contexts (35, 58, 60). Subsequently, legislative measures and humanitarian actions/interventions in favor of disabled individuals adopted the word “disability” as an umbrella term due to politics, rights-claiming initiatives and social “appearance” (61). Disability, in fact, does have a great economic and social impact among the poorest and most disadvantaged social groups in the Global North and South alike (62, 63) and those living in racialized contexts marked by state and police violence and the presence of gangs and endemic poverty (64). In addition to defining disability as a relational category, Friedner and Weingarten (65) have also pointed out how forms of bodily non-normativity are methods through which it is possible to analyze and describe processes of normalization and “habitus of ableism” (66) in society which are causes of marginalization, stigmatization and alterity for disabled individuals.

Within the broader discipline of present-day CDS, anthropology and its ethnographic methodologies have therefore allowed scholars to grasp relevant nuances on sociocultural and political meanings relating to various topics linked to disability (39). Among the topics tackled by anthropology, we can list the analysis of shared political and moral values about forms of bodily differences, life histories of people with disability (55, 67, 68), issues of citizenship and belonging, experiences of embodiment and subjectivity (26, 67) and religious ideas connected to bodily typologies (37, 39). Furthermore, ethnographic endeavors have provided timely and in-depth empirical data that represent the everyday lived experiences of people with disability in all their complexities. Anthropologists have also contributed to deconstruct dichotomic ideas of normality and abnormality in different cross-cultural contexts. Paraphrasing Ranganathan and Chetan (69), Disability Anthropology complements the scholarship and politics of CDS and complicates the figures of disability as a topic of investigation within medical humanities at large (38).

This review essay is structured around an exploration of anthropological epistemologies and ethnographic data collected and examined by scholars of Medical and Disability Anthropology. The next section summarizes the various stages through which disability has been investigated in anthropology and points out how forms of bodily alterity have been mainly experienced, conceived of and represented in areas of the Global South. However, such post- and decolonial ideas about non-normative bodies have been shaped by tensions with the global circulation of Euro-American ideas and practices through biomedical, global health and politico-humanitarian actions and interventions. Hence, the section after that, “multiple aspects of disability,” will highlight the various topics and themes researched by scholars in Disability Anthropology working in Global South settings and contexts. Finally, two sections will outline the theoretical and analytical relevance of conducting research on disability in societies outside the Global North as well as the epistemic and methodological implications of such endeavors.

## 2 Theories and methodologies of disability from the global north and south

Even before interest in disability widened in the early 1970s, a few pioneers in Social and Cultural Anthropology had explored such topics. Ruth Benedict conducted an analysis of epilepsy in 1934, and Jane and Lucien Hanks focused their attention on the sociocultural entanglements between physical “abnormalities” and social status in 1948 (39). Despite these early attempts, academic interest in disability began in earnest with civil rights movements around the 1960s, primarily in the US and the UK. Edgerton's study on the social reintegration of people with cognitive disabilities in 1967, Geyla Frank's analysis of Diane DeVries, an American woman who had no limbs due to a “congenital anomaly,” in 1982, and Joan Ablon's studies on people with dwarfism in the US are significant ethnographic and anthropological works on normativity, ableism and disability in Global North contexts (39). Developed in relation to and/or tension with analyses in the then-interdisciplinary arena of Disability Studies, these ethnographic works were the first to delve deeper into the subjective experiences of people with disabilities, examining their formation of networks of solidarity, demonstrating their capacity for agency, and exploring social practices around the social and political (re)affirmation of symbolic alter-states of “normality” beyond shared ideologies of able-bodiedness (39, 48, 70). These works are considered groundbreaking, as such scholars expanded the study of disability beyond the socio-therapeutic focus of the “medical model of disability” and highlighted that illness or suffering do not perfectly clarify its nature (26). More specifically, such interest was rooted in Euro-American social, economic, and political contexts characterized by phenomena such as generalized economic advancement, overspecialization, and capital division brought by industrialization and neoliberal economics. In Global North geographical areas, social norms and relationships built on neoliberal economic foundations and social structuration have resulted in a push toward ableism and behaviors that marginalize individuals with disabilities and non-normative bodies (19).

However, most people with disabilities live outside Euro-American geographical areas (31). As a result, anthropologists, sociologists, and scholars from the Global South have directed their attention to understanding how disability and other forms of bodily alterity are perceived, experienced, and lived by individuals belonging to diverse social categories and classes in these regions (26, 31, 37–39). These alternative conceptions of disability not only have broadened knowledge about different ways of experiencing bodily non-normativity in relation to social norms but also challenged prevailing Global North assumptions about social, economic, and political aspects connected to this matter (32). The growing interest in disability in anthropology and CDS has been further motivated by the expanding globalization of markets and commodities since the late 1970s. Disability gained prominence on the world political agenda starting in the 1980s, with the United Nations first declaring 1981 the “International Year of Disabled Persons” and then 1983–1992 the “United Nations Decade for Disabled Persons” (60).

From the 1990s to the early 2000s, as anthropologists and scholars in CDS have developed tensions and engagements, anthropological analysis has fallen within the disciplinary fences of Medical Anthropology (26). Besides criticizing practices of medicalization, some scholars (36) have looked at disability and definitions of bodily non-normativity by analyzing the implementation of reproductive technologies worldwide. For example, a proliferation of *in vitro* fertilization in Egypt and India has exacerbated social challenges facing couples with infertility issues that had been regarded as manifestations of social and cultural “impairment” (36). By emphasizing the sociocultural, political, spatial, and historical dimensions of the concept of disability, many studies have also demonstrated how conditions considered as non-normative in the Euro-American context are perceived as normal in other geographic regions including Nicaragua, Borneo, and various African countries (29, 35). Apart from the political influences, exerted by Global North countries on parts of the Global South, it is worth noting that the notion of disability either has not appeared yet or has emerged recently with the intensification of economic-political relations accelerated by globalization. In this regard, Ingstad and Whyte (35) highlight the absence of umbrella terms like “disability” or “disabled person” in many languages around the world to describe individuals with non-normative bodies. Instead, various descriptive terms exist to define specific forms of disability (35). One example is found in the ethnographic study by Matthew Kohrman (71) on the “bio-bureaucracy” surrounding the category of *canji* (disabled people) in China. While this term is seldom used in rural areas, it has become part of the everyday vocabulary used in major urban centers to describe the experiences of individuals with diverse disabilities. This shift occurred following the establishment of the “Chinese Federation for People with Disabilities” by the disabled son of a prominent national politician, which led to discussions on political and legal issues related to individuals with non-normative bodies. As Kohrman demonstrates, the term's proliferation has afforded political appearance to those labeled with it and catalyzed rights-advocacy initiatives and state-driven forms of economic support (71). In this ethnographic example, it emerges that the diffusion and/or imposition of the socio-political and legal category of “disability” in many Global South and North areas has been facilitated by top-down political interventions and state regulations (47, 56) that have changed existing types of community care (72).

The proliferation of the umbrella term and legal/political category of “disability,” along with other biomedical terms, beyond Global North contexts, has contributed to the (re)emergence of informal communities and networks of solidarity among people with disabilities. The formation and transformation of such groups can be traced back to the colonial era. These communities were often intertwined with the introduction of colonial biomedical and scientific endeavors in parts of the Global South territories. For example, groups of individuals with leprosy in India, Mali, and Tanzania arose from the intersection of sociocultural perceptions of the condition, colonial classifications, biomedical public health interventions and postcolonial categorizations (73–75). In these geographical areas, informal networks of solidarity among individuals with similar non-normative bodies are shaped by shared economic, employment needs (76, 77) as well as the presence of decolonial epistemics (78).

A similar finding in all these sociocultural experiences is the way people with disabilities navigate their sociality in local contexts where sociocultural norms differ from forms of individualism and “independent living” (35). Since disability and/or non-normative bodies are inherently relational, clearly the negative effects of disability stigma in societies of the Global North often stem from negative perceptions of social, economic, and political forms of “dependency”. Neoliberal notions of self-sufficiency, personal autonomy, and the significance of achieving economic and social independence through work and labor are key criteria against which “degrees of impairments” and disability are assessed. Consequently, values such as individuality, equality, and independence, which are often taken for granted in Global North contexts, clash with notions and aspirations of communitarian interdependence in other sociocultural contexts. When such ideas are promoted globally under various power dynamics, there is a risk of elevating them to universal ideas of (human) rights (26, 39). Although set in the Global North academic world, Murphy's foundational autoethnography (79) illustrates the marginalization and stigma faced by individuals with disabilities who cannot adhere to values of independence and individuality. Due to sudden paralysis caused by a spinal tumor, this US anthropologist and university professor personally experienced the ableism in the academic world and how bodily non-normativity stripped him of his social status (80).

In certain Global South contexts, cultural and socio-centric values associated with community and family membership can hold greater significance than individual capabilities and influence the idea of disability (35). An example of this paradigm is the concept of “debility” introduced by Julie Livingston (50, 81). Tswana notions of kinship and personhood emphasize the interconnectedness of individual bodies. As a result, emotions such as bitterness, anger, or jealousy can harm or alter the body. Livingston shows (50) how in Botswana disruptions in mental and/or physical capacities due to various disabilities and/or chronic illnesses are seen as the consequence of negative influences from other members of society. Therefore, “debility” describes how forms of bodily non-normativity result not solely from past colonial genealogies of exploitation as well as individual accidents but can also be linked to moral misdeeds and behaviors of individuals within the network of relationships surrounding the person with the condition. In other words, marginalization and social issues are not directed toward the individual but redistributed within the community. For instance, stigma can be directed at the single mother of a disabled child, whose disability is seen as the consequence of amoral sexual intercourse (81). However, anthropologists studying disability aim to avoid constructing false sociocultural and political dichotomies between “individualistic” groups in the Global North and “community-based” networks in the Global South. Instead, ethnographic studies have highlighted how disabled persons are able to navigate these complex sociocultural aspects in situational ways to either hinder or facilitate individual achievements and relational integration within a specific cultural context (35).

In summary, this section has shown how scholars of Medical and Disability Anthropology—as well as CDS researchers—amidst various types of epistemic divergences and/or collaborations-have shifted their focus from the experiences and perceptions of non-normative bodies in primarily Global North settings to contexts in the Global South. Such shifts have yielded new ethnographic data and insights on this topic. For example, research conducted in India, China, and Egypt has on the one hand revealed that ideas of normativity are socio-culturally and geographically bounded, and on the other that political discourses and narratives around disability have various local and global dimensions. Such new attention to the ontological existence of categories for defining sociocultural and temporal forms of bodily alterity leads to the exploration of various symbolic and material ways of describing disability. More specifically, anthropologists have also remarked that alternative notions for describing and experiencing non-normative bodies are also influenced by individuals' and groups' living conditions, as well as historical and political dynamics. The following section therefore delves deeper into the methodological and empirical contributions made by the anthropological analysis of disability to the study of experiences, representations and practices of individuals and groups with non-normative bodies with respect to shared norms.

## 3 Multiple aspects of disability

Previous research has revealed the multiple ways Global North biomedical and human rights definitions of disability have traveled and, through unbalanced politico-economic relationships and humanitarian/human rights' interventions, influenced various socio-cultural dynamics and definitions of non-normative bodies in Global South contexts. Furthermore, such studies have provided the broader research field of CDS with firsthand ethnographic data relevant to and analysis of how disabilities are experienced in geographical areas around the world. To avoid simplistic dichotomies between Global North and South contexts, the next subsections explore three aspects of disability in regions of the Global South, as they have been intercepted and studied by anthropologists and scholars of CDS in general.

In contrast to the entrenchment of “disability as a fixed category,” these aspects also epitomize enacted methodologies and epistemological attempts that offer novel ways to comprehend assumptions and experiences of “normalcy” (65). The selection of these three aspects therefore takes into consideration the characteristics previously identified by other scholars. Ginsburg and Rapp (37, 38) have clarified and critically summarized the global/local dynamics concerning disability rights and politics, the construction of alternative kinship frameworks, feminist and gender perspectives on difference, biopolitics, and the structural violence faced by individuals with non-normative bodies and the significance of material-symbolic infrastructures, technologies, and digital realms. In a similar vein, Devlieger (39) has directed attention to citizenship and belonging in the context of disability, to the impact of technology, and to the use of autoethnography, embodiment, and reflexivity as modalities through which individuals with disability articulate and scrutinize their own encounters and engagements with norms in society. Building on one of the first analyses of disability in sociocultural anthropology (35, 60, 82), other scholars have delved deeper into the intricate connections between disability and various forms of technology (e.g., social, material/instrumental, technical, etc.) in numerous African countries (83). Finally, Staples and Mehotra (26) have delineated the intricate and complex relationships between anthropology and disability studies and called for more attention to empirical data and theories from the South.

In the context of these empirical and theoretical developments in CDS and Medical and Disability Anthropology, the following sub-sections discuss three specific approaches that shed light on social, economic, political, gendered and historical domains that have been widely studied and/or should be further developed in relation to ideas and practices surrounding non-normative bodies in the Global South.

### 3.1 Phenomenological dimensions of non-normative bodies

In recent years, sociocultural and political analyses of disability in various geographical areas have been enriched by experiential and phenomenological perspectives on living with non-normative bodies in a given society (39). Several ethnographic works have highlighted how experiences of being-in-the-world with a non-normative body are determined not only by individual will but also shaped by power dynamics and sociocultural categorizations imposed by political changes, postcolonial dynamics, colonial histories, social institutions and individual economic status (47, 56, 71, 73, 74). For instance, communities for people with leprosy in India and Mali were formed at the intersection of sociocultural conceptions, biomedical categorization, and colonial classifications, resulting in emotional and social unity among individuals with similar non-normative conditions (73, 74).

Some discussions have focused on the biopolitical systems and their “paternalistic endeavors” (84) governing disabled bodies and the care provided to them; others have also investigated the phenomenological dimensions of disability. However, the latter are intrinsically linked to power dynamics, so anthropologists have begun to incorporate intersubjective perspectives and place greater emphasis on the “emic” character of their research. Reflexive field practices, autoethnography (79), and deep narrative approaches have been instrumental in broadening the understanding of disability as non-normative bodily experience (37, 39). Mattingly's paradigmatic studies (85, 86) have examined the relationship between everyday experiences of disability and the ethical-moral nuances within low-income African American families. Through an intersectional analysis, the scholar has explored how these individuals create networks of meaning and subjectivities and showed the importance of race, sex, gender, and social class in worsening issues related to disability in a neoliberal and market-driven society. Her subsequent research on African American families caring for children with chronic illnesses has more deeply explored the symbolic and material formation of “moral laboratories” representing metaphorical spaces in which these families transform everyday life and navigate economic, social, and political uncertainties (86).

Studies employing phenomenological and biopolitical approaches have been perceived as counteracting the “medical and social models of disability” (87). By merging subjective and social perspectives, these studies have reintegrated various forms of disabilities in their visceral, “enfleshed” and embodied experiences while examining the intersection between individual perceptions and political, medical, and religious aspects (26). For instance, Kathryn Geurts' research (88) among Anlo-Ewe speakers in southeastern Ghana exemplifies the epistemological novelty resulting from combining phenomenological and historical approaches. Geurts finds that experiences of disability among her research participants are intimately linked to a unique way of perceiving and imagining the sensory sphere. Instead of the Global North model of “five senses,” sensory capacity is attributed to bodily actions like balance and posture. The “vernacular” concept of *seselelame* expresses the sensations imprinted on and perceived by the body, flesh, or skin that carry moral and social meanings among the Anlo sociocultural group (88). Furthermore, this type of approach (50, 81) also provides the readers with post- and decolonial perspectives about disability.

Studies like Geurts's demonstrate the significance of phenomenological and sociocultural perspectives to understanding disability and its intersection with broader social, historical, and cultural contexts. By examining lived experiences and subjective realities, anthropologists have contributed to a more comprehensive understanding of disability as a relational, complex and multi-dimensional phenomenon in many Global South settings (26). This question is especially relevant in the present-day world, as various new technologies are changing the ways people with disabilities inhabit and participate in society and connect to other people (83). In various African regions and Global South settings, the adoption of various forms of technological devices—from crutches, wheelchairs, and prostheses to laptops and other digital tools—has changed the ways people with disabilities perceive their bodies, undergo processes of neoliberal and technological embodiment, and experience different community or social perceptions than in the past (89).

This first subsection has illustrated one of the most prominent aspects of the experiences of people living with disability: the sociocultural and political experiences of individuals with non-normative bodies, as well as the embodiments of their condition in relation to prevailing notions of normality in a particular society and the global circulation of symbols and ideas about their conditions. The study of disability has enabled scholars to comprehend how perceptions of non-normative bodies are invariably shaped and influenced by social, cultural, economic, political, gendered, racial, symbolic and historical factors. Consequently, phenomenological approaches that encompasses political dynamics, gender dimensions, affective relationships, and emotions have illuminated numerous perspectives for defining, conceptualizing, and representing disability in diverse global contexts beyond the confines of Global North epistemics.

### 3.2 Socio-political assemblages

Disability Anthropology has also paid attention to the understanding of non-normative bodies by exploring the diverse interactions and solidarity networks formed by individuals with similar institutionally-and-politically-classified types of disabilities and across various forms of non-normativity. The production of various kinds of relationships of dependence and solidarity with other disabled and/or able-bodied individuals is a related topic of such scholarly endeavor (90).

First, family and kinship networks are among the forms of relationships where ideas and practices about disability are (re)produced, challenged, and supported (39, 91, 92) and where forms of care and “activist affordance” are enacted (66). For example, in India the impact of non-normative conditions is primarily felt within the domestic sphere, leading to conflicts and the reshaping of family dynamics (91). Within these spaces, rewriting family kinship networks due to the presence of an individual with disability or a d/Deaf person becomes the norm and is the source of internal conflicts and readjustments (76). Similarly, in the United States the birth of a child with disability necessitates a redefinition of notions and ideas of normality and kinship and often causes a reformulation of relationships between parents and their children (92). Therefore, disability not only creates new forms of kinship but also challenges existing ones. Muslim migrant women with disability in Canada, for instance, have been described as resisting racialization and stigmatization while asserting their humanity through daily practices of resilience (93).

Identity-based communities are another important setting for the formation and transformation of ideas and practices related to disability. D/deaf communities in Japan, for example, prefer to define themselves as autonomous linguistic entities rather than solely as groups of people with disabilities or as d/Deaf people. By emphasizing their linguistic identity at the national level, they challenge social stigma and discrimination (94). Similarly, d/Deaf individuals in India participate in non-hearing support associations to better access the labor market and navigate legal obligations related to disability and employment (76). Sign languages, as a form of sociocultural expression, constitute ways of aggregation and protection from society's problems (95). Many of these deaf communities, as pointed out previously, do not want to be labeled as “disability groups” but as cultural minorities in both Global North and Global South sociocultural contexts (96, 97).

Furthermore, forms of formal and informal belonging to and membership in specific groups help individuals with disabilities gain entry to religious organizations (77) and pursue careers within state administration (71). In other words, Deaf communities in various geographic areas share common and similar experiences, stories, emotions, as well as cultural and linguistic traits (76, 94, 95). While cultural identification with deafness is expressed in the English-speaking world by capitalizing the word “Deaf,” the lowercase version of the term refers to the biomedical understanding of deafness in opposition to hearing. Given this differentiation, many people position themselves as d/Deaf to include both sociocultural and biomedically-constructed realities (39). Given the heterogeneity of ideas about and experiences of deafness, many researchers have disagreed with the imposition of a single political identity, preferring to emphasize that deafness could be classified as a set of linguistic and cultural—as well as political—characteristics attached to minorities (98). Instead of exploring Deaf communities, socialities, experiences and assemblages (95), Friedner (54) has recently also focused analytical attention on individuals and their “desire to become normal” (95) in their “disability worlds” (37). This examination of the techno-political and social landscapes of cochlear implants for deaf children in India reveals the ambivalence of various paths and modes of pursuing “sensory normality” (95).

The production of categorizations of disability by nation-states and international institutions has also influenced the formation of social networks around specific identity politics. Global political agendas, such as the “United Nations Decade for Disabled Persons,” have indirectly placed people with disabilities within specific politico-legal categories based on characteristics and socioeconomic backgrounds. In Greece, for instance, psychiatric reform led to the production of intangible values like “autonomy” and “individual responsibility” to secure rights for those with intellectual disabilities or cognitive-psychological issues (39, 99). Likewise, individuals affected by chronic illnesses and disabilities in post-socialist Ukraine identified themselves as “biological citizens” to access economic and biomedical aids after the Chernobyl disaster (47). The social battles waged by associations of people with disabilities due to spinal cord injury, again in Ukraine, represent another prime example of the forms of assistance and support implemented by such networks of organizations and activist groups (56). By adopting various creative strategies to be visible in the urban environment and asserting forms of “mobile citizenship,” Ukrainian activists have sought to raise state and public awareness (56). Along this line of research, the injured bodies of veterans in Turkey and at the Walter Reed National Military Medical Center in the US have been seen as becoming biopolitical surfaces on which right-wing values, ideas of masculinity, nationalist ideologies and moral ethical discourses are projected, discussed, criticized, reshaped and disrupted (100, 101).

Biopolitical and biosocial practices around conceptions of deafness are also manifested through the ways in which precise characteristics attached to non-normative bodies are experienced and described by the very same people who live with them. Friedner (76) reminds us of the importance of emphasizing how national contexts can materially produce different forms of deafness as well as encourage the promotion of “national sign languages.” The “civilizing project” of the Chinese state toward regional linguistic minorities, as in the Tibet Autonomous Region and Inner Mongolian Autonomous Region, is one example (102). Furthermore, Friedner has shown that during humanitarian works for the empowerment of people with deafness in Bangalore, American d/Deaf activists argued for the existence of a universal d/Deaf culture based on national and local socio-cultural specificities. In contrast, Indian d/Deaf students showed a certain disinterest in such universal values and were reluctant to participate in such social formations (37). Indeed, some of the Indian activists compared such activities to a kind of “deaf utopia” that would be difficult if not impossible to achieve (95). Structural violence and biopolitical/governmental practices toward various forms of non-normative bodies are not only concerned with the sociocultural, economic, and political dynamics of the material world but also extend to practices and discourses that concern material realities that are more difficult to perceive in real life.

Also, regarding deafness, Friedner (54) has recently investigated the epistemological possibilities offered by alternative modes of embodiment. As noted previously, her analysis of deaf people's multiple interactions with sound other than “hearing” describes non-normative practices related to socio-historical and cultural conceptions of “hearing” including experiencing physical phenomena such as vibrations. From an anti-biopolitical perspective, these practices enable them to recalibrate “phonocentric” models of language and even extend them to include forms of diversity she defines as “sensory sociality” (54).

The negotiation between state realities and support networks is crucial for understanding the formation and dissolution of social, economic, political, cultural, and affective ties. For example, in South Africa patient-physician encounters shape the allocation of social and health resources for people with disabilities (103). At the global level, the “United Nations Convention on the Rights of Persons with Disabilities” has provided a legal framework for local struggles for recognition and access to political and economic resources for people with non-normative bodies living in condition of political and economic deprivation. However, many states have ratified the convention without fully implementing it (104). Access to (inter)national rights and the advancement of specific “politics of deservingness” vis-à-vis state regulations and governmental measures for people with chronicity and disabilities (105) have thus been conditioned by the presence of local forms of patronage, racial politics (63) and social responsibility plans implemented by private entities and/or companies (76).

These struggles for rights and recognition often involve clashes between disability activists and institutional actors. Activists draw on universalist ideas of disability communities, while professionals rely on existing legislative models and claim technical expertise. This mismatch highlights the need for legislative measures that recognize disability as a (political) universal category while accounting for local perceptions and sociocultural models of corporeality (106). Moreover, these institutional actors take moral stances while activists rely on specific knowledge and ideas deemed “real.” This brief ethnographic example again underscores that a legislative, jurisdictional, institutional and, therefore, policy instrument that makes disability a universal legal category has yet to be created. Furthermore, legal and political discussions around the possibility of universalizing alternative local/indigenous concepts and descriptions of experiences of non-normative bodies are rarely considered in global discussions on this topic. The production of such instruments must consider the various ways in which disability and other alternative ways to define conditions of non-normative bodies are perceived within local and sociocultural models of corporeality, as well as by the various communities of disabled people (104).

As numerous studies reviewed in this second subsection have demonstrated, the dynamics of biosociality and governmentality encompassing similar categories and identity politics about various disabilities, as well as the stigma and marginalization imposed on individuals with non-normative bodies by the structures of able-bodied society, constitute two key factors in the formation of different types of groups, as well as in the development of dynamics of group belonging. It is not only state-driven categorizations, politico-institutional classifications and biomedical taxonomies of disability that play a role: technologies, biomedical interests, human rights advocacy, and legal propositions can also spark sentiments of belonging and (dis)belonging among people who share similar forms of bodily non-normativity.

### 3.3 Working activities and labor

Finally, many anthropologists have recently investigated work connected to disability and to the neoliberal global economy in which non-normative bodies are immersed (23, 76, 77, 107–110).

Historians of disability in Euro-American contexts like Stiker (58) have refuted earlier ideas and shown how work and remunerative labor were human activities that indirectly produced the category of disability. Through social movements in the Global North, this category spread to Global South localities—such as Botswana and Sierra Leone—through humanitarian actions in post-conflict contexts and situations of deprivation (81, 87). Conditions of unemployment or labor exploitation imposed on Global South settings by capitalist and colonial economic policies have produced and/or exacerbated several forms of social impairment related to disability (63, 104). Neither humanitarian development's emphasis on creating educational capital and market-driven working capacities in disabled individuals (23, 107, 108) nor global and national and humanitarian-driven entrepreneurial activities for people with disabilities (76, 109, 111) have changed these disadvantageous and unfavorable living conditions. Hence, their long-term impact has been significant (112).

Disability therefore has become an integral part of work and can also serve as a means for labor creation and income generation. This understanding is derived from numerous ethnographies that explore the utilization of bodies “productive” endeavors and settings. Two cases from India and the Democratic Republic of Congo (DRC) can shed light on this aspect. In one, Staples (74) demonstrates how a community of people with leprosy in the Bethany Colony in India perceives begging as a form of work and its members carefully present their disability and use their bandages or visible wounds to perform it and attract the attention of passers-by. In the other, Devlieger (77) describes a group of individuals with physical disabilities who traverse the capital city of Kinshasa in the DRC, soliciting money through begging. Their “work” is distinctive in that they produce pages from humanitarian documents or engage in “NGO paperwork” to convince the public about the seriousness of their situation. These instances highlight the agentic utilization of disability by individuals as a way to acquire economic resources and support. Moreover, these studies have also emphasized how people with non-normative bodies forge collective identities around income-generating activities with people with similar conditions and form relationships with “able-bodied” ones (68).

At a different level, Friedner (76) has documented the employment of deaf individuals in the business outsourcing sector in India. Through her ethnographic work, the individuals she portrays highlight how specially designated jobs for people with disabilities, including deaf individuals, symbolically emphasize the distinction between non-normative bodies and physical characteristics that align with societal norms and the rules set up by the Indian state. Friedner's essay also underscores that these politically-imposed-specialized occupations for deaf workers reinforce specific biases about deafness within Indian society. For example, she points to the perception that deaf workers are more obedient and reliable than their non-disabled counterparts. In this regard, deaf workers are described as being able to appreciate deaf-friendly environments and the supportive attitudes of their employers and the Indian government (76).

In addition to Staple and Friedner's observations regarding the interplay between work and disability, other anthropologists working in Global South contexts have also pointed out that what Global North societies perceive as non-employment is considered work or a job in different sociocultural settings. Biehl's (49) ethnography of individuals facing various health issues in areas characterized by socioeconomic and political uncertainty and deprivation illustrates this point. One of his research participants, Catarina, engages in daily activities that can be considered forms of symbolic and social labor, such as writing poems, a sort of dictionary, and a diary. The categorization of this as a work stems from her pursuit of survival and livelihood within an environment marked by social abandonment. This specific ethnographic study once again emphasizes the futility of creating rigid dichotomies between able-bodied individuals seen as productive and disabled individuals condemned to be labeled as unable to work within the confines of the neoliberal regime, which perpetuates their exploitation (108).

Finally, anthropological exploration of the intersection between work and disability has shed light on how working activities and diverse forms of employment can reinforce and reshape intersubjective affective bonds and the sense of shared experience. Scholars in CDS and disability anthropology have emphasized that work can unveil possibilities of interdependence and community supports, which tend to prevail in many Global South contexts (25). The use of various technologies at work has been observed to foster networks of solidarity among people with disabilities: for example, among those who use wheelchairs and other mobility aids to get around and transport cargo in Uganda (82). In numerous settings, practices of shared experiences within work extend beyond the performance of specific tasks to encompass strategies of acquiring technical skills and fostering social connectivity to cope with social and economic challenges (23).

This third subsection has therefore highlighted anthropologists' recent exploration of disability through the lens of labor and recognition of people with non-normative bodies as active agents within the workforce. Beyond post-Fordist neoliberal industrial and productive contexts, primarily in the Global North or under its influence and control, many of them now challenge the portrayal of individuals with disabilities merely as “unproductive” or “dependent” people outside the realm of capitalist production or “targets” of humanitarian and human rights initiatives. On the contrary, recent ethnographic contributions have shown the intricate connections between different forms of work and experiences of disability in diverse contexts in both the Global North and Global South (23, 76). Within this subsection, the concept of work emerges as a pathway to social participation and an opportunity for individuals with disabilities to contribute actively in contexts outside the Global North. Anthropological studies have, in fact, shown how disabled individuals may strategically employ their “physical” appearances to generate income and assume the role of “humanitarian subjects” (68, 74, 77). While notions of labor and work for people with disabilities, as well as able-bodied individuals, encompass activities beyond the generation of capital, other scholars have also explored how new business models draw upon stereotypical notions of disability to frame and commodify disability in multiple ways (110).

From these considerations, it becomes evident that examining the socio-political, economic, and historical implications of disability and work, including the daily activities of individuals with disabilities, provides insights into their individual and collective perspectives on work. Moreover, such analysis enriches the understanding of the biopolitical and phenomenological dimensions of what work signifies for people with disabilities residing in diverse social contexts and geographic regions.

## 4 Discussion

CDS is a complex and multidimensional field of research that brings together scholars from various academic disciplines, activism, and social work sectors. Its scope extends to exploring the social, political, economic, racial, gendered, and historical constructions of non-normative bodies across diverse global contexts. Additionally, this interdisciplinary arena aims to analyze ideas, practices, and narratives generated by institutional and humanitarian infrastructures working on disability-related topics in both the Global North and South. This review has specifically focused on the ethnographic data and debates presented by scholars rooted in Social and Cultural Anthropology, particularly in the sub-fields of Medical Anthropology and Disability Anthropology/Anthropology of Disability. Such limitation outlines, however, creative tensions and engagements between anthropologists and CDS scholars in generating empirical and theoretical knowledge about disability. In considering the intersectional themes and epistemic goals of CDS researchers, this article has critically explored the rich ethnographic materials that illuminate sociocultural understandings, lived experiences, economic possibilities, work activities, and historical genealogies of various indigenous and global-local categories of non-normative bodies. Using methods such as participant observation, focus groups, reflections on the researcher's ethnographic positionality, and diverse epistemological understandings, anthropologists have been able to gather multiple forms of ideas of disability and able-bodiedness in Global Southern and Northern settings. These insights, combined with media, historical, visual, and political materials analyzed by scholars from other disciplines within the interdisciplinary arena of CDS, have challenged Euro-American ideas, narratives, and institutional practices regarding disability and its socio-political classifications. In this endeavor, scholars in “crip theory” and CDS play a crucial role in anthropological discussions on disability, as queer and decentered perspectives turn out to be useful in critically examining previous conceptions of disability in academia and activism (17, 27).

The three aspects of the social and anthropological study of disability highlighted in this review article pertain to various domains and states of the sociocultural lives of disabled people in many societies: (1) experiences and politics, (2) belonging, kinship and citizenship, and (3) work and labor. These three dimensions underscore the importance of incorporating diverse global perspectives and expanding the research scopes of present and future anthropological studies on disability conducted by scholars in Social and Cultural Anthropology as well as in CDS. However, the three aspects/dimensions highlighted in this review article do not fully capture the complexity involved in the critical analysis of disability experiences, lives, and issues. Matters, ideas, and practices related to sexuality, same-sex sexualities, queerness/queering, masculinity, femininity, and their intersections with disability as well as decolonial understandings of bodily non-normativity can also contribute to the analysis of disability and provide further insights into its multiple genealogies (27). Research on these themes offers valuable empirical data on how specific societies perceive the connections between gender issues and non-normative bodies.

While there has been a recent special issue on the intersection of disability and technologies (83), further studies in other African and Global South regions could shed light on new engagements with technologies and digital worlds in relation to disability (113). Moreover, it is essential to explore which technologies are newly developed or transported from Global Northern localities and adopted in or adapted to new urban or rural environments through trade and humanitarian interventions. Another neglected topic concerns the experiences of BIPOC, decolonial, and indigenous individuals living with and experiencing non-normative bodies, either in contrast to globalized, human rights, and Global North ideas and practices about disability (18, 20–24, 114). For example, the “retheorization of disability studies through the employment of theories embedded in African Renaissance” may offer a solution for decentering theories and practices around non-normative bodies (115). Exploring this focus and its “research complexities” (116) could illuminate new ways of conceptualizing and presenting ideas regarding the body, social norms, notions of normality, and more. As highlighted by Ginsburg and Rapp (38), further studies and research may additionally investigate the “disabled ecologies” (117) that address the complex and impactful relationships between environmental justice and experiences of environmental pollution.

Despite their contributions, neither Social and Cultural Anthropology nor Medical and Disability Anthropology can comprehensively address the innumerable and complex sociocultural, historical, political, and economic facets of disability and non-normative bodies in various areas of the world. To truly engage with this complex subject, a more thorough collaboration with CDS scholars and collaborative efforts from other disciplines within and outside the borders of anthropology are imperative. Fields such as Gender Studies, Black Studies, Critical Global and Public Health, and Critical Pedagogy are essential to exploring the multifaceted aspects of disability and fostering a comprehensive understanding of such complex realities. Additionally, it is crucial that scholars devote more attention to critiquing ideas of biomedicalization and rehabilitation and engage more deeply with CDS scholarship.

## 5 Conclusion

Emerging from a critical standpoint rooted in the “social model of disability,” CDS has investigated how social, cultural, economic, political, gendered, and historical circumstances—as well as subjective experiences—influence and reshape ideas, practices, and discourses surrounding non-normative bodies and disability. While CDS scholars aim to examine issues of (neo)coloniality, gender dynamics, and ableism, their studies have primarily focused on Global North contexts.

Recognizing that “disabling experiences are universal, yet simultaneously shaped by specific circumstances” (39), Social and Cultural Anthropology, Critical Medical and Disability Anthropology provide valuable perspectives and empirical data from geographical areas and sociocultural contexts located in both the Global North and South. Through personal engagements and observational fieldwork, these scholars can unravel social dynamics surrounding people with non-normative bodies, explore the complexity of economic and political dimensions of disability, and bring nuances to the analysis of indigenous, post- and decolonial systems of knowledge regarding ideas of alterity and “normality/normalcy,” as well as expand on etiological and epistemic ideas and practices related to non-normative bodies. In so doing, fruitful collaborations with other disciplines outside anthropology are extremely useful and enriching.

This analysis and critical summary of the latest research in the fields of Medical and Disability Anthropology has therefore demonstrated how Global South contexts offer infinite possibilities and rich examples of lively experiences alongside apparently social impairments—or rather, sociocultural, historical, economic and political constructions, representations, and manifestations of such imagined and material constraints.

## Footnotes

^1^For analytical purposes, the present analysis will often employ the term disability. However, I take into account the many scholarly critiques of its Anglo-American Eurocentrism. Thus, I also use the less connoted and more generic terms “diverse/non-normative body-minds” interchangeably with it.

^2^As signaled by Hall (1), CDS and CDT refer both to “a diverse, interdisciplinary set of theoretical approaches” that examine disability as a socio-cultural, historical, symbolic, political phenomenon. The present article uses the term CDS in preference to CDT as it indicates the presence of both theoretical and methodological approaches in the study and exploration of disability-related topics in various geographical areas.

^3^Following Wolf-Mayer’s discussion (12) about the use of the term “bodyminds” in disability studies, I prefer to utilize “non-normative body/ies” when referring to disabled people/people with disability to avoid suggestions of ableism and eugenics and emphasize “people in bodies with livable lives” (12).

^4^I use the conventional terms “Global South” and “Global North” to delineate macro-geopolitical and geographical areas with their histories, political dynamics and social configurations. However, such geographical and geopolitical scales are no more precise than in the past due to the presence of complex, manifold and multi-centered power global and regional dynamics. Furthermore, as highlighted by Dados and Connell (34), “North-South terminology, then, like core-periphery, arose from an allegorical application of categories to name patterns of wealth, privilege, and development across broad regions” that do not correspond to the complexity of the present-day world. Therefore, readers should be aware of the profound limitations of such terminology.
